# A Study on the Application of Convolutional Neural Networks to Fall Detection Evaluated with Multiple Public Datasets

**DOI:** 10.3390/s20051466

**Published:** 2020-03-06

**Authors:** Eduardo Casilari, Raúl Lora-Rivera, Francisco García-Lagos

**Affiliations:** Departamento de Tecnología Electrónica, Universidad de Málaga, ETSI Telecomunicación, 29071 Málaga, Spain; raul.lora@uma.es (R.L.-R.); fgl@uma.es (F.G.-L.)

**Keywords:** fall detection system, accelerometers, body sensor networks, classification algorithms, convolutional neural networks, machine learning, wearable sensors

## Abstract

Due to the repercussion of falls on both the health and self-sufficiency of older people and on the financial sustainability of healthcare systems, the study of wearable fall detection systems (FDSs) has gained much attention during the last years. The core of a FDS is the algorithm that discriminates falls from conventional Activities of Daily Life (ADLs). This work presents and evaluates a convolutional deep neural network when it is applied to identify fall patterns based on the measurements collected by a transportable tri-axial accelerometer. In contrast with most works in the related literature, the evaluation is performed against a wide set of public data repositories containing the traces obtained from diverse groups of volunteers during the execution of ADLs and mimicked falls. Although the method can yield very good results when it is hyper-parameterized for a certain dataset, the global evaluation with the other repositories highlights the difficulty of extrapolating to other testbeds the network architecture that was configured and optimized for a particular dataset.

## 1. Introduction

Due to the growing life expectancy and the social changes in the traditional family structure, the population of seniors that live alone in their homes has notably increased during the last few decades. In this context, falls are a major risk for the quality of life and the autonomy of the elderly.

According to the studies reported by the World Health Organization (WHO) [1], falls represent the second leading cause of accidental deaths around the world, producing a particularly high morbidity among people aged 65 and older. For those aged over 80 residing in community settings, the percentage of those persons that experience at least one fall per year climbs to 50% [2], with 40% of them suffering recurrent falls [3]. In the USA, the annual number of fall-related injuries is expected to reach 3.4 million in 2020 and 5.7 million by the year 2030 [3]. As it refers to the economic impact on the sustainability of national health systems, the global medical costs attributable to falls in 2015 totaled about $50.0 billion [4].

Aid response time is a key element to prevent the most serious potential consequences of the comorbidities and disabilities linked to falls. Consequently, the study of systems for the automatic recognition of falls has become an important research topic in the fields of telemedicine and human activity recognition during the last ten years.

The objective of Fall Detection Systems (FDSs) is to continuously monitor the movements of a certain user (or patient) with the aim of transmitting an alarm notification (text message, phone call, etc.) claiming assistance to a remote observation point whenever a fall is suspected. FDSs must be carefully designed to discriminate falls from other routines or ADLs (Activities of Daily Living) so that the number of both unnoticed falls and false alarms (ADLs misidentified as falls) is minimized.

In spite of the variety of existing solutions to the problem of fall detection, FDSs are usually categorized into two groups [5,6,7]: context-aware (vision and/or ambient based) and wearable detectors. In context-aware systems, falls are recognized by processing the signals collected by environmental sensors (such as cameras, depth sensors, microphones, vibration sensors, etc.) located in the vicinity of the subject to be tracked. Hence, the operation of a context-aware architecture is confined to the particular zone (e.g., room, nursing home) where the sensors are deployed and configured. In this zone, the alteration of factors such as the lighting, the disposition of the furniture, or the presence of unexpected elements (occlusions, pets, falling objects, other individuals, spurious sounds, etc.) may impact heavily on the effectiveness of the detection decision [8]. Furthermore, in the case of using audiovisual equipment, the patients may feel their privacy compromised.

On the other hand, wearable systems permit monitoring the patient’s movements by means of one or several transportable sensors (mainly accelerometers, but also gyroscopes, and much less frequently, magnetometers or ECG sensors), which are fixed to the clothes or attached to the body through elastic bands.

Wearable FDSs can be easily implemented on smartphones as these popular gadgets natively embed inertial sensors. Otherwise, if the sensing capabilities of the phone are not leveraged, external sensors can also connect with a smartphone via a low-power wireless standard (such as Bluetooth Low Energy) with a view to benefit from the long range connectivity (Wi-Fi, 4G/3G) of these personal devices. Thus wearable FDSs offer a cost-effective alternative to track the movements unequivocally linked to a certain user practically, without any geographical or location restriction. The increasing capacity of wearables to put into operation sophisticated detection algorithms has also fostered the interest in this typology of FDSs within the research community.

Falls are generically and ambiguously defined by the WHO as events that “result in a person coming to rest inadvertently on the ground or floor or other lower level” [1]. Due to the complex dynamics and the broad variety of the types of falls, fall detection algorithms based on machine learning techniques yield much more accurate results than those obtained by ‘thresholding’ strategies [9,10], which simply compare a certain variable or groups of variables (e.g., the acceleration magnitude) with one or several preset thresholds or limit values to produce the detection decision. 

In the domain of machine learning, different classes of architectures based on artificial neural networks such as Recurrent Neural Networks (RNNs) [11,12,13] have been successfully employed as the movement classifier of a FDS. Similarly, Convolutional Neural Networks (CNNs) have also been recently proposed as a promising technology for those HAR (human activity recognition) systems [14,15] and wearable FDSs that process the data gathered by inertial sensors [16,17,18].

CNNs are formed by a sequence of processing layers interrelated through neurons. Unlike other machine learning techniques commonly employed in wearable FDSs (such as Support Vector Machine or k-Nearest Neighbors), CNNs allow modeling the underlying structures of large datasets without requiring human guidance as they are capable of identifying those internal features that optimize the representation of the data with different layers of abstraction [19].

A central issue still under discussion in the study of fall detection algorithms is their evaluation. On account of the evident complexity of testing the FDS in a real scenario with actual falls suffered by elderly patients, fall detection algorithms are massively evaluated by the literature against databases of inertial measurements. These data are typically collected by a group of volunteers that transport inertial sensors during the systematic emulation of falls and ADLs executed as a function of a preconfigured testbed.

In almost all initial studies on FDSs, the resulting evaluation datasets were not made publicly available so that they could not be re-utilized by other authors to compare new proposals. This lack of specific benchmarks was partially remedied over the past years, during which several repositories designed for the evaluation of FDSs have been released. Thus, an increasing use of public datasets as benchmarking tools has been clearly detected in the recent related literature. However, although existing databases strongly differ in many aspects (sampling rate and range of the sensors, number and characteristics of the experimental users, emulated movements, length of the samples, etc.), in the vast majority of the works, the detection methods are parameterized and tested by taking into account only a single dataset. Thus, it is legitimate to question whether the results obtained by these studies for a particular dataset can be extrapolated when other test samples are considered.

In this paper, we evaluate the capability of a CNN to perform as the movement classifier in a wearable FDS. The hyper-parameters of the deep learning architecture are initially designed to optimize the detector performance when a specific dataset is utilized as the evaluation framework. Then, the same architecture is trained and tested with the other 13 datasets. The analysis shows the huge variability of the quality metrics, which deeply depend on the utilized repository.

The rest of this paper is organized as follows. Section 2 revises the existing datasets, while Section 3 comments on the configuration of the CNN (input features, layers, dimensions). Section 4 presents the numerical results systematically obtained for the different datasets when the same detection method is applied. Finally, the conclusions are recapitulated in Section 5.

## 2. Revision and Selection of Public Datasets

Due to the inherent complications of gathering inertial measurements of actual falls suffered by older people, most works have evaluated their proposals for FDSs by creating a testbed in which a set of volunteers transporting one or several inertial sensors systematically execute a predetermined number of activities. These preconfigured activities usually include typical ADLs (such as sitting, walking, running, climbing stairs, etc.) as well as some types of mimicked falls (which are normally carried out on a mat or padded surface to avoid injuries). Unfortunately, in many studies, these measurements obtained in the testbed are not made publicly available to be exploited by the research community to compare new proposals. To overcome this drawback, different datasets have been published (especially during the last four years) as benchmarking tools for cross-comparison of detection algorithms.

Table 1 presents a comprehensive list of the authors, reference, institutions, and year of publication of these datasets. Ahmed et al. [20] presented another repository (generated by 140 subjects and intended for assessing fall risk), which was not considered in this analysis as it only includes five falls.

All of these datasets comprise the measurements collected by the inertial sensors worn by the selected volunteers during the preconfigured experiments. The number of obtained samples and considered typologies of the emulated ADLs and falls, the duration of the traces as well as the basic characteristics of the participants (number, gender, and age range) are described in Table 2.

Table 3 summarizes, in turn, the type and basic properties (sampling rate, range) of the sensors employed to generate the repositories. The table also indicates the corporal position on which the sensor was located or attached during the experiments (see [21] for a further comparison of some of these datasets). As can be observed from the table, although there are cases where up to seven sensing positions have been considered, most datasets include only a single measuring point. In all cases, the sensor embeds at least an accelerometer and less often, a gyroscope, a magnetometer, and/or an orientation sensor.

These available repositories are being increasingly considered by the recent literature on algorithms for FDSs to evaluate the effectiveness of the detection process. Table 4 lists those studies that have employed public datasets in order to test neural algorithms aimed at detecting falls in wearable systems. The table itemizes the number and name of the utilized repositories as well as the quality metrics achieved by the neural detection methods (mainly sensitivity and specificity, or alternatively, accuracy or AUC (area ander the receiver operating characteristic curve). Table 4 shows that the evaluation of the method in most studies (12 out of 17) was limited to a single dataset. Only three works validated their proposals against more than two datasets. The work by Khojasteh et al. [42] made use of four databases, but two of them (DaLiac [43] and Epilepsy [44] repositories) only included ADLs (which only allows for evaluating the capacity of the system to avoid false alarms). The interesting dataset from the FARSEEING project [35], also used in that study, consists of the traces obtained from 300 real world falls captured by monitoring a population of hundreds of older people for several weeks. However, only 22 samples of that repository were available (under request to the project managers). The FARSEEING dataset was also taken into consideration by one of the two studies that employed three datasets: the work by Mauldin in [39], which introduced two similar databases (known as Smartwatch and Notch) collected with wrist sensors (a smartwatch and an external IMU-Inertial Measurement Unit-, respectively). Apart from the problems related to the difficulties of detecting falls with a wrist worn device, these databases, also used by Santos in [18], incorporate a moderate number of fall events that may hamper a thorough and systematic assessment of the effectiveness of the detector.

From the previous analysis, we can conclude that the use of several benchmarking datasets has not been a major concern in the literature focused on wearable fall detection systems. However, before being applied, all machine learning strategies for fall detection (including neural methods) need to be configured by setting the values of a not-negligible number of parameters (e.g., the number and nature of input features). In most cases, these values are heuristically selected, presumably as a function of the results obtained with a set of testing samples extracted from a very particular dataset. As no other database is utilized, the study of the capability of the configured network to detect falls under conditions different from those in the reference dataset (sensor model, sampling rate, typology of ADL and falls, etc.) actually remains unaddressed. Furthermore, except for some works such as that by Yuwono [45] (which considers an outgroup dataset), the samples applied to test the system were always acquired from the same experimental subjects that provided the training (and validation) samples.

In this regard, by using three repositories (tFall, DLR, and MobiFall), Medrano et al. have already shown in [46] that the performance of a FDS noticeably decreased when the machine learning algorithms were evaluated on a dataset different from that employed for training. In this work, we show that even when the algorithm is trained and tested with data of the same datasets and users, the performance of the same method may vary dramatically depending on the contemplated repository. With this in mind, we selected SisFall as our reference dataset to parameterize the CNN in order to maximize the performance metrics. Then, we analyzed the resulting network configuration when it was trained and tested with other 13 different datasets (ticked with a check mark in Table 1).

We opted to use SisFall [36] as the basis of our analysis as it is one of the most employed datasets in the literature (see Table 4). In addition, it was generated by one of the largest sets of participants (38 subjects including 19 males and 19 females) with the widest age range (19–75 years). SisFall contains a significant volume of traces (2707 ADLs and 1798 mimicked falls) with a duration (between 10 s and 180 s per movement, with a mean value of 15 s) long enough to apply different analysis strategies. The nature of the emulated activities also exhibits a noteworthy variety: 19 categories of ADLs (ranging from jogging or stumbling to basic movements such as sitting down) and 15 different types of falls (generated as a function of the direction of the fall and the initial user’s position). In the testbed deployed to collect the SisFall samples, the volunteers transported the sensing on a belt. The waist is believed to be a good (and ergonomic) position to characterize the user’s movements [60] as it is near the gravity center of the body and not strongly associated to the individual mobility of a limb (such as the wrist or the ankle). For comparison purposes, we also selected those datasets where the data were collected on the waist (or at least, on the upper part of the thigh). In order to keep a common evaluation framework, in those cases where the traces were gathered with several sensors simultaneously located on several parts of the body (e.g., Erciyes or UMAFall datasets), the analysis focused on the data obtained on the waist and the data from the other sensors were not utilized.

Nevertheless, we have to remark that the appropriateness of investigating fall detection systems with falls emulated by young healthy participants on a cushioned surface is still a controversial topic out of the scope of this work. In this respect, Klenk found remarkable differences between the mobility patterns of emulated and real-life [61] falls. In contrast, after analyzing the dynamics of actual falls endured by elderly people, Jämsa et al. concluded in [62] that intentional and real life falls exhibited analogous characteristics.

## 3. Configuration of the Convolutional Neural Network

### 3.1. Selection of the Input Features

The selection of the input features is a key design decision for the performance of machine learning strategies.

In most practical implementations of FDS, the detector is expected to be (at least partly) implemented on a sensing mote with heavy hardware resource limitations of the battery and computation power. Thus, to facilitate the real-time operation of the wearable, input features should be derived from the data collected by the sensors without requiring any complex preprocessing of the signals. In this respect, the architectures of the CNNs are particularly suited to learn the internal structure of the signals directly from the raw sensor data without any previous heuristic extraction of input features. Accordingly, we propose to directly feed the input of the CNN with the raw inertial measurements provided by the repositories instead of using other parameters computed from the data (extreme values, statistical moments, autocorrelation, time between ‘peaks’ or ‘valleys’ of the signals, wavelet or discrete Fourier transform coefficients, frequency domain features, etc.). As long as some datasets include other types of measurements, in this paper, we focused on the analysis of the triaxial accelerometry signals (which are the basis for fall detection in most wearable FDSs existing in the literature).

Falls are normally associated with one or several sudden upsurges of the acceleration magnitude caused by the impacts of the body against the ground [63]. Hence, our analysis will be concentrated on a time interval of fixed duration around the instant in which the maximum value of the acceleration magnitude is detected, implicitly assuming that in the case of the sequence with a fall event, the fall has occurred during this interval.

The acceleration magnitude or Signal Magnitude Vector ($SMV_{i}$) for the *i*-th sample can be directly computed from the values measured by the triaxial accelerometer:

(1) $$SMV_{i}=\sqrt{{\left|A_{x_{i}}\right|}^{2}+{\left|A_{y_{i}}\right|}^{2}+{\left|A_{z_{i}}\right|}^{2}}m/s^{2}$$

where *A_xi_*, *A_yi_*, and *A_zi_* define the three components of the acceleration vector for that *i*-th sample in the direction of the *x*, *y*, and *z*-axis, respectively. These components are periodically measured by the tri-axial accelerometer embedded in the smartphone and the external sensors.

The acceleration peak or maximum of the SMV (S*MV_max_*) is defined as:

(2) $$SMV_{max}=SMV_{t_{o}}=max\left\{SMV_{i}∶i\in \left[1,N\right]\right\}$$

where *N* indicates the length (number of samples) of the analyzed trace while *t_o_* is the index of the sample at which the acceleration peak is located.

Following the typical fall pattern, a “free fall” period (in which the acceleration magnitude tends to be zero) normally precedes the impact against the floor. Furthermore, the dynamics of a fall is usually characterized by brusque modifications of the body orientation, which are reflected in abrupt changes in the sequence of the three acceleration components. In this context, the typical duration of fall has been reported to span between 1 s and 3 s [64]. Thus, in order to capture the most significant elements of the dynamics of a fall, we propose setting an observation window of up to ±2.5 s around the instant *t_o_* (four different window sizes will be considered). Hence, the CNN is fed with the raw data collected by the accelerometer during that period.

**Figure 1:**
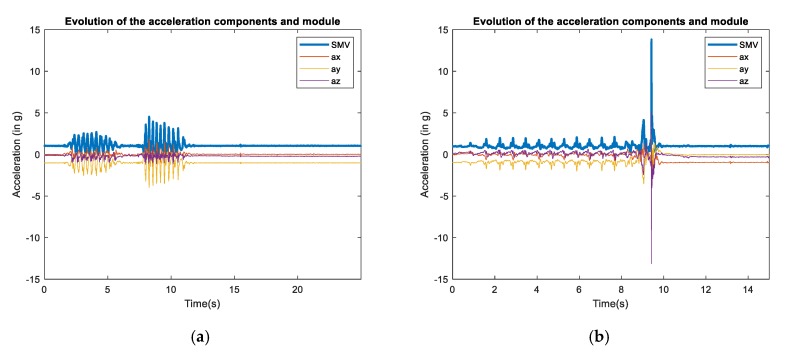
Snapshot of the progress of the acceleration components (*Ax, Ay, Az*) and magnitude (SMV) for two samples in the SisFall dataset: (**a**) An ADL (walking upstairs and downstairs quickly) and (**b**) a fall (forward fall while walking caused by a trip).

Figure 1 illustrates an example of the evolution of both the acceleration components and magnitude for a particular ADL (climbing and descending stairs rapidly) and a forward (mimicked) fall caused by a trip.

**Figure 2:**
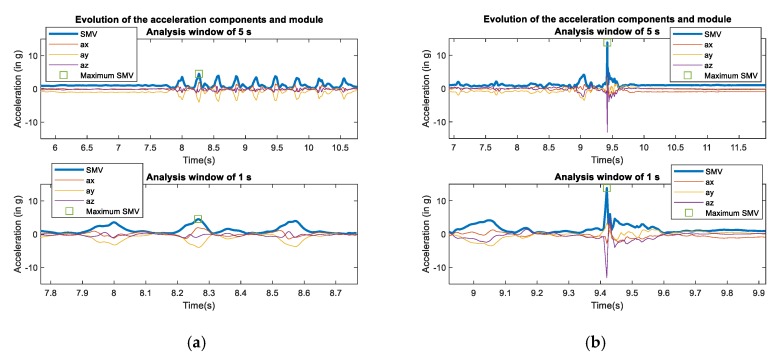
Detail of the progress of the acceleration components (*Ax, Ay, Az*) and magnitude (SMV) of the examples of Figure 1 for two different observation windows (±0.5 s and ±2.5 s) around the detected maximum of the SMV. (**a**) ADL; (**b**) Fall.

Figure 2 represents the time series that will be analyzed by the CNN after extracting the values corresponding to the observation window around the peak magnitude (for two window sizes: 1 and 5 s).

The sub-figures, in which the value $SMV_{max}$ is indicated with a square marker, clearly show that window sizes larger than 5 s are not required to apprehend the variability of the mobility patterns during the fall. In our analysis, we will take into consideration two alternative variants for the input sequences of the CNN:

1. The series of the acceleration modules (*SMV_j_*) computed from the samples collected during the observation window:
  (3) $$SMV_{j}\;\forall j\in \left[t_{o}-\frac{T_{W}}{2}f_{s},t_{o}+\frac{T_{W}}{2}f_{s}\right]$$
  where *f_s_* indicates the sampling rate of the accelerometer and *T_W_* represents the duration (in seconds) of the window.
2. As the second set of input features, we directly consider the series of the triaxial acceleration components ($A_{x_{j},}A_{y_{j},}A_{z_{j}}$) obtained from the sensor:
  (4) $$A_{x_{j},}A_{y_{j},}A_{z_{j},}\;\forall j\in \left[t_{o}-\frac{T_{W}}{2}f_{s},t_{o}+\frac{T_{W}}{2}f_{s}\right]$$

In the case of SisFall traces, as the sampling frequency is 200 Hz, a 5 s window encompasses 1001 values of the acceleration magnitudes and 3003 input features when the triaxial components are employed.

### 3.2. Structure of the CNN

The basic objective of a neural network is to autonomously discover and implement a relationship between a set of fixed-size input features (here, the accelerometer data) and a known set of fixed-size output labels (here, a binary decision of 0 or 1, depending on the movement type, ADL or fall).

Classical multilayer perceptrons (MLPs) present full neuron connectivity between contiguous layers (which dramatically increases the number of synaptic weights). In contrast with this repetitive structure of neurons of MLPs, CNN are composed of specialized layers conceived for different purposes. Thus, some elements are only responsive to a particular zone or ‘region’ (for images) or interval (for time series) of the original input data. Benefitting from this ‘clustering’, the initial (convolutional) layers in an CNN are in charge of learning and extracting the ‘features’ that characterize the different pattern types to enable the discrimination at the final stage.

In MLPs or under other machine learning strategies, the features (or internal representation of the raw data) required to feed the classifiers must be ‘manually’ or heuristically selected. Hence, the performance of the system strongly relies on the expertise of the designer [19].

In a CNN, the learned features from a certain group of neurons in a layer (computed through simple but nonlinear combinations of the neuron inputs) are the inputs for some neurons of the following convolutional layer. Therefore, the high-level abstraction of the raw data was carried out in an automatic way and with multiple levels of representation. To this end, in the convolutional layers, every neuron convolves the received data with a set of adjustable kernels (or filters) of a predefined size. The coefficients of these filters are adjusted during the training phase to optimize the representativeness of the features. After the convolution, the resulting values are passed through a non-linear activation function. In our case, we used rectified linear unit (ReLU), which is widely extended to deploy CNNs. ReLU can be easily computed as a ramp function: *f(z) = max (z,0)* [19].

In order to reduce and compact the feature vectors produced by the convolutional filters into a ‘down-sampled feature map’, we utilized pooling layers after the convolutional layers. As a result, every element or neuron in the pooling layer is capable of condensing the information generated by a region of neurons of the previous layer. In particular, we employed the popular max-pooling filters, which simply extract the highest value of the input region.

After the sequential feature extractors, a final classifier is required to produce the final discrimination decision based on the global features learned by the closing convolutional layer. In our scheme, although other conventional machine learning strategies could have been considered, we used an architecture comprising one fully connected layer and a *softmax* function, which normalizes the weighted input feature vector into two values, which describes the probabilities of detecting an ADL or a fall. The final classification of the movement is simply based on the maximum of these two probabilities.

To implement the CNN, we utilized MATLAB [65] scripts by leveraging the Deep Learning MATLAB Toolbox^TM^ [66]. To operate with these scripts, the CNN is directly fed with an equivalent ‘image’ of (1 × *width*) ‘pixels’, where the term *width* describes the number of acceleration samples contained in the observation window around the acceleration peak (1001 or 3003, for the case of SisFall dataset). Thus, the system was trained to categorize the ‘images’ as two different output types (ADL or fall).

### 3.3. Training Procedure

In order to prevent overfitting during the training process, the original traces of the employed datasets were divided into three independent sets of samples: training, validation, and test sets, following the typical ratio of 60% (for training), 20% (validation), and 20% (testing). The repositories were randomly split, but preserving the same proportion of falls and ADLs in the three groups.

The validation set was used to assess the performance of the network after a certain number of iterations or epochs in which the CNN was trained with the training sample group. As the training progresses, the error (or loss) committed when classifying the validation samples is expected to gradually decrease. Thus, the process continues until this validation loss stops decreasing and keeps increasing for a predetermined number of attempts (‘validation patience’). This fact indicates that the network is beginning to overfit the training data and, consequently, that the learning phase needs to be concluded. To reduce the effects of overfitting in deep learning, we also employed two common complementary techniques: dropout and L2 Regularization, aimed at avoiding co-adaptations on training samples and minimizing the sum of the values of the weight coefficients, respectively.

**Figure 3:**
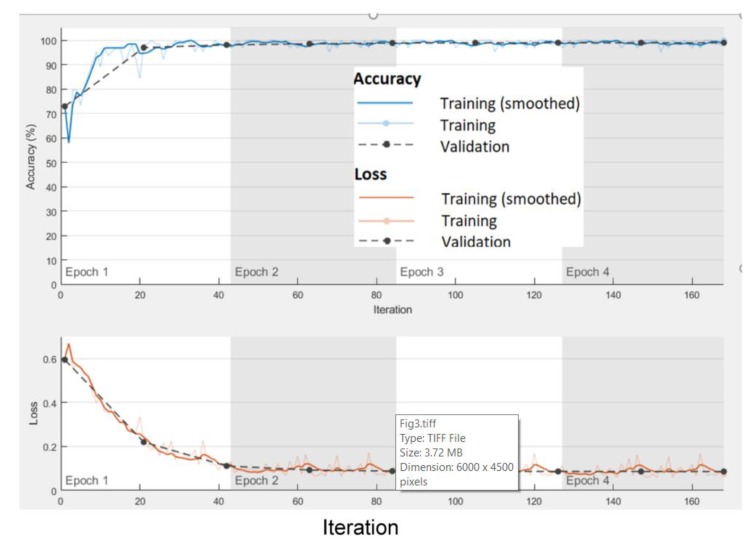
Training progress: training and validation accuracy.

We established a validation patient of three epochs. If this limit is not exceeded, the learning phase stops after 20 epochs. Figure 3 illustrates the rapid convergence of the accuracy and loss for the training and validation sample sets during the training process when the SisFall dataset is employed.

In short, Table 5 summarizes the final characteristics and hyper-parameters of the utilized CNN as well as the procedures considered for the training phase. Through an initial phase of hyperparameter optimization and a systematic evaluation of the architecture, the network was dimensioned and hyper-parameterized to maximize the performance metrics achieved with the SisFall repository.

As previously commented, the complexity of a CNN is determined by the typology and number of layers. A basic architecture with just one or two convolutional layers may be sufficient to learn the features in a small set of unsophisticated data. However, more layers are normally required to detect complex patterns in datasets as those used in our study. Therefore, as can be seen in Table 5, the CNN consisted of four consecutive feature extractor and one final classifier. Every feature extraction layer includes one convolutional layer, followed by one batch normalization layer, a nonlinear ReLU activation function, and one down-sampling max pooling layer (except for the final convolutional layer, which does not incorporate the max pooling operation). Likewise, the final classifying layer is formed of one fully-connected layer, one *softmax* function, and one final classifying step. The training process uses the cross-entropy loss function.

## 4. Numerical Results

After training the neural system, the performance of the trained CNN was evaluated by computing the efficacy of the detection decisions when the network was fed with the independent set of test samples of every dataset.

To evaluate the capacity of the CNN to discriminate falls from ADLs, we calculated three traditional quality metrics to characterize the performance of binary classifiers: the sensitivity (*Se*); hit rate or recall, which describes the ability to recognize falls; the specificity (*Sp*) or selectivity, which portrays the effectiveness of the FDS to prevent false alarms (i.e., ADLs misinterpreted as falls); and accuracy (*Acc*), as a global measurement of the system efficiency.

These quality metrics (defined as percentages) can be straightforwardly computed as:

(5) $$Sensitivity\left(\%\right)=100\cdot \frac{TP}{FN+TP}$$

(6) $$Specificity\left(\%\right)=100\cdot \frac{TN}{FP+TN}$$

(7) $$Accuracy\left(\%\right)=100\cdot \frac{TP+TN}{TN+FN+TP+FP}$$

where *TP* and *TN* define the number of ‘True Positives’ and ‘True Negatives’ (i.e., falls and ADLs that have been adequately identified), respectively. Similarly, *FP* and *FN* describe the number of ‘False Positive’ and ‘False Negatives’ (ADLs and falls that have been misidentified).

The results obtained when the network was trained and tested with the reference dataset (SisFall) are available in Table 6 (also presented in [67]).

The table displays the quality metrics achieved for an observation window of ±2.5 s for the two alternative input signals (the SMV and the triaxial acceleration components). The results seem to indicate that the effectiveness of the detector (namely the sensitivity) noticeably improves when the 3-axis signals are utilized. This could be justified by the fact that, when compared to the acceleration magnitude, triaxial components offer a better insight about the sudden changes of the body orientation provoked by the falls, which could facilitate the CNN in the detection decision.

In any case, the obtained results (with both specificity and sensitivity near 99%) were better than those published by other studies on FDS that employed the same SisFall dataset as a benchmarking tool [36,52,56,68,69] and in which a specificity and a sensitivity superior to 0.98 were not attained simultaneously.

However, the performance of the system considerably worsens if we extend the evaluation to the other datasets. For that purpose, we also selected those existing databases that incorporate samples obtained with an accelerometer located in a similar position (waist or, if not possible, thigh).

Table 7 and Table 8 shows the results for the 14 considered repositories when the SMV and the triaxial acceleration components are respectively considered as the input features (the results for reference SisFall dataset are highlighted in bold font). The tables include the metrics achieved for four different observation windows (±0.5 s, ±1 s, ±1.5 s, and ±2.5 s around the acceleration peak). In two cases (UniMiB and DLR), the short duration of the samples prevented the study for the highest values of the window size.

We have to remark that in all cases, the CNN architecture was trained, validated, and tested with data extracted from the same dataset (no cross-validation between different datasets was contemplated) and followed the same procedure as that applied to the SisFall dataset. By the same token, for each repository, the number of inputs of the CNN were dimensioned, taking into account the sampling rate used to generate the dataset and the desired observation window (no sub-sampling or over-sampling of the dataset measurements was performed). In this way, we evaluated the capability of the deep learning architecture to self-adapt to the different conditions under which the traces were collected in each repository.

From the tables, we can conclude that the performance of the system visibly depends on the employed benchmark. No clear trend can be deduced about the behavior of the detector: in some cases, an acceptable specificity (higher than 95%) was achieved at the cost of an inadmissible sensitivity while for other datasets, the detector seems to prioritize the sensitivity (with independence of the number of ADLs and falls included in the repositories). In some cases, the accuracy is even worse than that achieved with a random classification of the movements with balanced data (50%). No conclusions can be drawn either from the analysis of the importance of the observation window or from the election of the input features.

This ‘erratic’ performance of the detector can be justified by the huge variability of the factors that affect the generation of the mobility patterns: parameters of the sensor (range, sampling rate), typology of the emulated movements, characteristics of the experimental subjects or configuration of the scenario of the simulations (pads, mattresses, etc.).

Consequently, in opposition to the procedure commonly followed by most works in the literature, we consider that it is essential to evaluate any proposal on a FDS against diverse databases to state that the accuracy of the classifier has been validated.

In any case, this issue is just another example of the existence of a global problem associated with the research on fall detection systems: the lack of commonly accepted methodology to evaluate and benchmark the new proposals on FDSs existing in the literature. This lack of consensus, which has been highlighted and discussed by different authors [70,71,72,73], does not only affect the employed datasets, but also other important operational aspects of the evaluation policy (performance metrics, availability of the code of the proposed algorithms, etc.). Thus, the problem of the proper selection (number and typology of movements, participants, etc.) of the datasets should be approached under a general procedure to generate a consensual framework that eases the comparison of FDSs.

## 5. Conclusions

The last decade has witnessed a considerable number of research efforts that propose new algorithms to detect falls based on the signals captured by wearable inertial sensors.

This work has evaluated the effectiveness of a Convolutional Neural Network (CNN) to discriminate falls and ADLs (Activities of Daily Living) from datasets containing accelerometry signals.

In contrast to other machine learning strategies, the system reduces the need of the required preprocessing of the signals and avoids the ‘handcrafted’ selection of input features as it takes advantage of the ability of CNNs to automatically extract knowledge from complex raw time series. Thus, the CNN is directly fed with the signals collected by an accelerometer. In particular, the analysis of the CNN focuses on the acceleration samples gathered during an observation window around the moment where a certain peak in the acceleration magnitude is detected.

The performance of the classifier was initially evaluated by using one of the largest public datasets of movements with emulated falls. The achieved performance metrics (with specificity and sensitivity over 98%) revealed that the effectiveness is augmented when the three acceleration components (instead of the acceleration magnitude) were used as input features by the CNN. Then, the obtained architecture was trained and tested with the samples of 13 other public databases, collected with an accelerometer in a similar position but in different testbeds.

Results show that the dataset employed as a benchmark dramatically impacts the performance, which could be justified by the variability of the sensors and the configuration of the tests deployed by the testbeds of the different repositories. These results challenge the methodology usually followed by the related literature, by which the proposed algorithms are evaluated against one (or at the most two) datasets.

By using a deep learning architecture, capable in principle of automatically extracting the most representative features of the training patterns, we have shown the difficulty of extrapolating the results achieved for a dataset when the same fall detection architecture is evaluated with samples of the same nature, but collected in different scenarios (employed sensors, sampling rate, type of movements, characteristics of the experimental subjects, etc.). Therefore, these results at the very least question the effectiveness of many machine learning mechanisms that achieve excellent performance metrics (with sensitivities and specificities close to 100%) when they are trained and validated with samples recorded in a very particular experimental setup. This conclusion is particularly relevant if we take into account that in a realistic application of a FDS, it is very unlikely that the system can be trained with samples of actual falls of the target user.

In any case, future studies should analyze in detail whether these limitations in the extrapolation capability of the FDS can be resolved if more sophisticated configurations of the CNN are tested or if other machine learning mechanisms (either the traditional feature engineering-based methods or other neuronal mechanisms such as LSTM (Long-Short Term Memory Networks) are considered.

To the best of our knowledge, this is the first proposal that utilizes up to 14 different public datasets to assess the effectiveness of a FDS. Further studies should be devoted to defining a framework to characterize the quality and representativeness of the existing datasets, so that a commonly accepted procedure to evaluate fall detection systems could be defined.

## Figures and Tables

**Table 1:** List of the existing public datasets intended for the study of wearable fall detection systems. (* Note: √ indicates the datasets employed in this study).

Dataset	Ref.	Authors	Institution	City (Country)	Year	*
DLR	[22]	Frank et al.	German Aerospace Center (DLR)	Munich (Germany)	2010	√
LDPA	[23]	Kaluza et al.	Jožef Stefan Institute	Ljubljana (Slovenia)	2010	
MobiFall MobiAct	[24] [25]	Vavoulas et al.	BMI Lab (Technological Educational Institute of Crete)	Heraklion (Greece)	2013 2016	√ √
EvAAL	[26]	Kozina et al.	Department of Intelligent Systems, Jozef Stefan Institute,	Ljubljana (Slovenia)	2013	
TST Fall detection	[27]	Gasparrini et al.	TST Group (Universita Politecnica delle Marche)	Ancona (Italy)	2014	√
tFall	[28]	Medrano et al.	EduQTech (University of Zaragoza)	Teruel (Spain)	2014	√
UR Fall Detection	[29]	Kępski et al.	Interdisciplinary Centre for Computational Modelling (University of Rzeszow- UR-)	Krakow (Poland)	2014	
Erciyes University	[30]	Özdemir & Barshan	Department of Electrical and Electronics Engineering (Erciyes University)	Kayseri (Turkey)	2014	√
Cogent Labs	[31]	Ojetola et al.	Cogent Labs (Coventry University)	Coventry (UK)	2015	√
Gravity Project	[32]	Vilarinho et al.	SINTEF ICT	Trondheim (Norway)	2015	
Graz UT OL	[33]	Wetner et al.	Graz University of Technology	Graz (Austria)	2015	√
UMAFall	[34]	Casilari et al.	Dpto. Tecnología Electrónica (University of Málaga)	Málaga (Spain)	2016	√
FARSEEING	[35]	Klenk et al.	FARSEEING Consortium (SENSACTION-AAL European Commission Project)	Five hospital or scholar centers in Germany and one university in New Zealand	2016	
SisFall	[36]	Sucerquia et al.	SISTEMIC (University of Antioquia)	Antioquia (Colombia)	2017	√
UniMiB SHAR	[37]	Micucci et al.	Department of Informatics, Systems and Communication (University of Milano)	Bicocca, Milan (Italy)	2017	√
SMotion	[20]	Ahmed et al.	Department of Computer Science (University of Karachi)	Karachi (Pakistan)	2017	
IMUFD	[9]	Aziz et al.	Injury Prevention and Mobility Laboratory (Simon Fraser University)	Burnaby (BC, Canada)	2017	√
DU-MD	[38]	Saha et al.	Department of Electrical and Electronic Engineering (University of Dhaka)	Dhaka (Bangladesh)	2018	
SmartFall & Notch datasets	[39]	Mauldin et al.	Department of Computer Science, Texas State University	San Marcos (TX, USA)	2018	
UP-Fall	[40]	Martínez-Villaseñor et al.	Facultad de Ingeniería (Universidad Panamericana)	Mexico City (Mexico)	2019	√
DOFDA	[41]	Cotechini et al.	Department of Information Engineering (Università Politecnica delle Marche)	Ancona (Italy)	2019	√

**Table 2:** Basic characteristics of the experimental subjects and the emulated movements for the different datasets (n.i.: not indicated).

Dataset	Number of Subjects (Females/Males)	Age	Number of Types of ADLs/Falls	Number of Samples (ADLs/Falls)	Duration of the Samples (s)
DLR	19 (8/11)	[23–52]	15/1	1017 (961/56)	[0.27–864.33] s
LDPA	5 (n.i.)	n.i.	10/1	100/75	Up to 300 s
MobiFall MobiAct	24 (7/17) 57 (15/42)	[22–47] [20–47]	9/4 9/4	630 (342/288) 2526 (1879/647)	[0.27–864.33] s [4.89–300.01] s
EvAAL	1 (n.i.)	n.i.	7/1	57 (55/2)	[0.162–30.172]
TST Fall detection	11 (n.i.)	[22–39]	4/4	264 (132/132)	[3.84–18.34] s
tFall	10 (3/7)	[20–42]	Not typified/8	10909 (9883/1026)	6 s (all samples)
UR Fall Detection	6 (0/6)	n.i. (over 26)	5/4	70 (40/30)	[2.11–13.57] s
Erciyes University	17 (7/10)	[19–27]	16/20	3302(1476/1826)	[8.36–37.76]
Cogent Labs	42 (6/36)	[18–51]	8/6	1968 (1520/448)	[0.53–55.73] s
Gravity Project	2 (n.i.)	[26–32]	7/12	117 (45/72)	[9.00–86.00] s
Graz UT OL	5 (n.i.)	n.i.	10/4	2460 (2240/220)	[0.18–961.23] s
UMAFall	17 (7/10)	[18–55]	8/3	531 (322/209)	15 s (all samples)
FARSEEING	15 (8/7)	[56–86]	0/22	22 (0/22)	1200
SisFall	38 (19/19)	[19–75]	19/15	4505 (2707/1798)	[9.99–179.99] s
UniMiB SHAR	30 (24/6)	[18–60]	9/8	7013 (5314/1699)	1 s (all samples)
IMUFD	10 (n.i.)	n.i.	8/7	600(390/210)	[15–20.01]
DU-MD	10 (4/6)	[17–20]	8/2	3299 (2309/990)	[2.85–11.55]
Smartfall	7 (n.i.)	[21–55]	4/4	181 (90/91)	[0.576–16.8]
Smartwatch	7 (n.i.)	[20–35]	7/4	2563 (2456/107)	[1–3.776]
UP-Fall	17 (8/9)	[18–24]	6/5	559(304/255)	[9.409–59.979]
DOFDA	8 (2/6)	[22–29]	9/9	432 (120/312)	1.96–17.262

**Table 3:** Position and characteristics of the sensor used in the different datasets. Note: A: Accelerometer, G: Gyroscope, O: Orientation measurements, M: Magnetometer. SP: Smartphone.

Dataset	Number of Sensing Points	Captured Signals in Each Sensing Points	Positions of the Sensing Points	Type of Device	Sampling Rate (Hz)	Range
DLR	1	3 (A, G, M)	Waist (belt)	1 external IMU	100	±5 g (A) ±1200°/s (G) ±75 μT (M)
LDPA	4	Position (x,y,z coordinates)	Right ankle, Left ankle, Waist (belt), Chest	4 external IMUS (tags)	10	Tens of meters
MobiFall & MobiAct	1	3 (A, G, O)	Thigh (trouser pocket)	1 smartphone	87 (A) 100 (G,O)	±2 g (A) ±200°/s (G) ±360° (O)
EvAAL	2	1 (A)	Chest, right Thigh	2 external IMUs	50	±16 g (A)
TST Fall detection	2	1 (A)	Waist, Wrist	2 external IMUs	100	±8 g (A)
Erciyes University	6	3(A, G, M)	Chest, Head, Ankle, Thigh, Wrist, Waist	6 external IMUs	25	±16 g (A) ±1200°/s (G) ±150 μT (M)
tFall	1	1 (A)	Alternatively: Thigh (right or left pocket), Hand bag (left or right side)	1 smartphone	45 (±12)	±2 g (A)
UR Fall Detection	1	3 (A)	Waist (near the pelvis)	1 external IMU	256	±8 g (A)
Cogent Labs	2	2 (A, G)	Chest, Thigh	2 external IMUs	100	±8 g (A) ±2000°/s (G)
Gravity Project	2	1 (A)	Thigh (smartphone in a pocket) Wrist (smartwatch)	1 smartphone 1 smartwatch	50	±2 g (A) ±16 g (A)
Graz UT OL	1	2 (A, O)	Waist (belt bag)	1 smartphone	5	±2 g (A) ±360º (O)
UMAFall	5	3(A, G, M)	Ankle, Chest, Thigh, Waist Wrist	1 Smartphone 4 external IMUs	100 (SP) 20 (IMUs)	±16 g (A) ±256°/s (G) ±4800 μT (M)
FARSEEING	1	2 (A,G)	Waist or Thigh	1 external IMU	100	±6 g (A) ±100°/s (G)
SisFall	1	3 (A, A, G)	Waist	1 sensing mote with two accelerometers and a gyroscope	200	±16 g (A1) ±8 g (A2) ±2000º/s (G)
UniMiB SHAR	1	1 (A)	Thigh (left or right trouser pocket)	1 smartphone	50	±2 g (A)
IMUFD	7	3(A, G, M)	Chest, Head, Left ankle, Left thigh, Right ankle, Right thigh, Waist	7 external IMUs	128	±16 g (A) ±2000°/s (G) ±800 μT (M)
DU-MD	1	1 (A)	Wrist	1 external IMU	33	±4 g (A)
Smartwatch	1	1 (A)	Wrist (left hand)	Smartwatch (MS Band)	31.25	±8 g (A)
Notch	1	1 (A)	Wrist	1 external IMU	31.25	±16 g (A)
UP-Fall	5	2 (A, G)	Ankle, Neck, Thigh (pocket) Waist, Wrist	5 external IMUs	14	±8 g (A) ±2000°/s (G)
DOFDA	1	4 (A, G, O, M)	Waist	1 external IMU	33	±16 g (A) ±2000°/s (G) ±800 μT (M)

**Table 4:** Employed datasets and obtained results in other works that propose neural detectors.

Work	Ref.	Number of Employed Datasets & Names of the Datasets		Sensitivity	Specificity
(Poorani et al., 2012)	[47]	1	Not specified dataset obtained from UCI machine learning repository	91%	n.i.
(Cheng & Jhan, 2013)	[48]	1	LDPA	52%−78%	95.6%−99.47%
(Rashidpour et al., 2016)	[49]	1	MobiFall	100%	100%
(Özdemir & Turan, 2016)	[50]	1	Erciyes University	94.20%−96.27% (Accuracy)	
(Vallabh et al., 2016)	[51]	1	MobiFall	89.23%	81.43%
(Carletti et al., 2017)	[52]	2	tFall & SisFall	91.2%−94.4%	95.4%−98.1%
(Jahanjoo et al., 2017)	[53]	1	MobiFall	91.89%−97.29%	98.7%−100%
(Khan & Taati, 2017)	[54]	2	DLR & Cogent Labs	70−95%	65%−90%
(Khojasteh et al., 2018)	[42]	4	UMAFall, DaLiAC, Epilepsy & FARSEEING	83.33%−100%	80.13%−84.18%
(Lisowska et al., 2018)	[16]	1	tFall	AUC (Area Under the Curve): (0.582−0.904)	
(Mauldin et al., 2018)	[39]	3	FARSEEING, Smartwatch and Notch	89%−100%	70%−99%
(Musci et al., 2018)	[55]	1	SisFall	85.78%−97.18%	94.14%−99.01%
(Nguyen et al., 2018)	[56]	1	SisFall	98.26%	99.62%
(Theodoridis et al., 2018)	[57]	1	UR Fall Detection	96.67%	100%
(Chelli & Patzold, 2019)	[58]	1	Cogent Labs	96.8%−99.11%	100%
(Santos et al., 2019)	[18]	3	UR Fall Detection, Smartwatch and Notch	22.73%−99.72%	87.50%−100%
(Wisesa & Mahardika, 2019)	[12]	1	UMAFall	23.6%−100%	74.1%−97.6%
(Yacchirema et al., 2019)	[59]	1	SisFall	AUC (Area Under the Curve): (0.582−0.904)	

**Table 5:** Architecture and training hyper-parameters of the employed CNN.

Training algorithm	Stochastic Gradient Descent Momentum
Error function	Cross-entropy loss function
Maximum number of training epochs	20
Mini-batch size (to estimate the gradient of the loss in every iteration)	64 training instances
Validation frequency	1 epoch
Validation patience	3
Tecniques to prevent overfitting	Cross-validation, L2 Regularization and dropout layers
Initial learning rate:	0.0001
Layers activation functions	ReLU (hidden layers) and *softmax* (output layer)
Number of convolutional feature extraction layers	4
Sub-layers for every feature extraction layer	4 (1 convolutional, 1 normalization, 1 ReLU and 1 max pooling layers)
Number of filters for each convolutional layer	16 (1st layer), 32 (2nd), 64 (3rd), 128 (4th)
Filter size (for all convolutional layers)	1 × 5
Size of zero-padding	2 samples
Stride	1 × 1 (“non-strided”)
Pool size of the max-pooling layer	1 × 5
Classification layers	1 fully-connected layer, 1 softmax layer and 1 final classifier

**Table 6:** Results of the detection system for the SisFall dataset (observation window *T_W_* = ±2.5 s).

Input Signal	Performance Metric		
	Sensitivity	Specificity	Accuracy
SMV	96.34%	95.44%	96.97%
3-axis signals	98.91%	98.69%	98.78%

**Table 7:** Comparison of the obtained performance metrics for the 14 datasets and the four different observation windows (*T*_W_) around the peak when the acceleration magnitude (SMV) was used to feed the convolutional neural network (the results for the reference dataset -SisFall- are marked in bold). Note: * Some observation windows could not be applied to these datasets due to the short duration of the samples.

	Duration of the Observation Window Around the Peak											
	T_W_ = ±0.5 s			T_W_ = ±1 s			T_W_ = ±1.5 s			T_W_ = ±2.5 s		
Dataset	Se	Sp	Acc	Se	Sp	Acc	Se	Sp	Acc	Se	Sp	Acc
Cogent Labs	0.00%	100.00%	77.75%	0.00%	100.00%	75.43%	2.38%	99.69%	75.08%	0.00%	100.00%	70.73%
DLR *	0.00%	100.00%	93.65%	0.00%	100.00%	96.99%						
Erciyes University	94.29%	94.17%	94.23%	97.56%	91.03%	94.69%	97.21%	93.02%	95.30%	96.77%	95.14%	96.05%
GRAZ UT OL	0.00%	100.00%	80.72%	0.00%	100.00%	86.75%	0.00%	100.00%	80.72%	0.00%	100.00%	77.11%
MOBIACT	56.72%	91.07%	80.24%	31.11%	94.83%	74.59%	31.54%	95.59%	76.00%	50.85%	78.03%	68.62%
MOBIFALL	69.09%	94.74%	82.14%	96.49%	81.82%	89.29%	96.77%	58.00%	79.46%	94.74%	77.27%	87.13%
**SisFall**	**91.74%**	**95.27%**	**93.90%**	**91.23%**	**96.46%**	**94.34%**	**90.40%**	**96.77%**	**94.12%**	**96.34%**	**95.44%**	**96.97%**
tFall	26.76%	99.64%	92.52%	62.12%	99.39%	96.01%	72.57%	99.44%	96.65%	80.00%	99.24%	97.39%
TST Fall Detection	96.88%	90.48%	94.34%	89.66%	95.83%	92.45%	92.59%	84.62%	88.68%	85.19%	84.00%	84.62%
UMAFall	0.00%	94.00%	76.42%	4.00%	97.96%	78.86%	0.00%	100.00%	81.30%	0.00%	98.92%	74.80%
UniMiB SHAR *	71.71%	97.53%	91.09%									
IMUFD	37.78%	60.32%	50.93%	43.59%	53.62%	50.00%	10.00%	86.76%	58.33%	0.00%	100.00%	67.59%
UP-Fall	92.86%	98.21%	95.54%	89.80%	100.00%	95.54%	94.55%	96.49%	95.54%	89.80%	98.41%	94.64%
DOFDA	96.30%	87.10%	92.94%	98.33%	92.00%	96.47%	98.41%	72.73%	91.76%	60.47%	26.67%	46.58%

**Table 8:** Comparison of the obtained performance metrics for the 14 datasets and the four different observation windows (*T*_w_) around the peak when the triaxial components of the acceleration magnitude were used to feed the convolutional neural network. (the results for the reference dataset -SisFall- are marked in bold). Note: * Some observation windows could not be applied to these datasets due to the short duration of the samples.

	Duration of the Observation Window around the Peak											
	T_W_ = ±0.5 s			T_W_ = ±1 s			T_W_ = ±1.5 s			T_W_ = ±2.5 s		
Dataset	Se	Sp	Acc	Se	Sp	Acc	Se	Sp	Acc	Se	Sp	Acc
Cogent Labs	0.00%	100.00%	80.00%	0.00%	99.61%	72.29%	0.00%	100.00%	75.38%	0.00%	100.00%	75.96%
DLR *	0.00%	100.00%	92.06%	0.00%	100.00%	94.58%						
Erciyes University	98.06%	87.92%	93.47%	96.64%	92.72%	94.84%	91.53%	92.13%	91.81%	92.88%	95.58%	94.08%
GRAZ UT OL	90.00%	23.29%	31.33%	64.29%	18.84%	26.51%	25.00%	41.27%	37.35%	7.69%	97.14%	83.13%
MOBIACT	20.00%	98.60%	72.71%	49.26%	95.50%	80.71%	44.53%	95.49%	79.06%	70.83%	88.16%	83.28%
MOBIFALL	100.00%	9.43%	56.76%	100.00%	51.61%	72.97%	98.39%	44.90%	74.77%	94.23%	85.71%	90.10%
**SisFall**	**96.74%**	**95.50%**	**96.00%**	**98.64%**	**99.63%**	**99.22%**	**98.56%**	**98.92%**	**98.78%**	**98.91%**	**98.69%**	**98.78%**
tFall	0.00%	100.00%	90.14%	0.00%	100.00%	89.72%	40.32%	99.80%	94.72%	61.57%	99.90%	96.10%
TST Fall Detection	96.30%	0.00%	49.06%	100.00%	31.82%	71.70%	84.00%	75.00%	79.25%	76.67%	72.73%	75.00%
UMAFall	29.03%	73.91%	62.60%	11.54%	88.66%	72.36%	0.00%	100.00%	84.55%	0.00%	100.00%	82.11%
UniMiB SHAR *	66.47%	96.40%	89.02%									
IMUFD	0.00%	100.00%	69.44%	0.00%	100.00%	62.96%	11.90%	77.27%	51.85%	0.00%	98.46%	59.26%
UP-Fall	78.18%	22.81%	50.00%	96.08%	59.02%	75.89%	82.98%	60.00%	69.64%	91.49%	53.85%	69.64%
DOFDA	100.00%	5.00%	77.65%	100.00%	0.00%	76.47%	100.00%	0.00%	74.12%	100.00%	0.00%	78.08%
